# The Relationship Between Sickle Cell Disease and Sudden Onset Sensorineural Deafness

**DOI:** 10.7759/cureus.9413

**Published:** 2020-07-27

**Authors:** Elabbass Abdelmahmuod, Mohamed A Yassin, Mohanad Ahmed, Elrazi Ali

**Affiliations:** 1 Internal Medicine, Hamad Medical Corporation, Doha, QAT; 2 Hematology and Oncology, Hamad General Hospital, Doha, QAT

**Keywords:** scd, sickle cell disease, sickle cell anemia, hemoglobin sickle cell, deafness, sensorineural hearing loss

## Abstract

Sickle cell anemia (SCA) is a hereditary hemoglobin (Hb) disorder associated with a very specific molecular lesion, which is the exchange of glutamic acid for valine in the sixth residue of the Hb beta chain, originating the S Hb. It is characterized by intermittent episodes of vascular occlusion and end-organ damage.

Progressive organ damage may affect any organ with brain, eyes, pulmonary, hepatobiliary, spleen, genitourinary, and the musculoskeletal systems being the most commonly involved and reported. Other complications of the disease that have not been well described or studied include cranio-orbital syndromes, oropharyngeal syndromes, periodontal disease, and otologic syndromes.

Considering the vaso-occlusive nature of sickle cell disease (SCD), the potential for auditory damage is not unexpected. However, the incidence of subjective hearing impairment among SCA is very low and and little is known about the relationship between SCA and hearing loss. Here we report a 43-year-old female with SCA who presented with sudden bilateral hearing loss and generalized body ache and admitted as a case of sensorineural deafness with vascular crisis; she received IV fluid and analgesia and improved after five days from the therapy.

## Introduction

Sickle cell disease (SCD) is an inherited hematological disorder, which affects red blood cells (RBCs). Normal RBCs can squeeze through small blood vessels. However, sickled or crescent-shaped RBCs become stiff and sticky. These changes lead to difficulty in passing through small blood vessels. SCD is the most common among African, Indian, Middle Eastern, Mediterranean, and Latino descent [1-2].

The typical presentation of SCD

When sickle-shaped cells block small blood vessels, less oxygen reaches that part of the body, leading to widespread organ and tissue damage. Such damage causes familiar complications of SCD, which include painful episodes in the arms, legs, chest and abdomen, recurrent acute chest syndrome, a pneumonia-like symptom due to lung tissue damage, severe anemia, stroke, and painful, prolonged erections called priapism in males [3].

Sickle cell disease sometimes associated with endocrinopathies like severe hypogonadism could be due to primary hypogonadism, recurrent testicular infarction, and zinc deficiency. Besides testicular dysfunction, sickle patients may also have abnormalities in other sex organs like prostate gland and seminal vesicles, both affected in ejaculation volume and may lead to infertility [4].

Blood transfusion has been a traditional modality for the treatment of acute and chronic complications of SCD. It improves blood flow by decreasing the sickling of Hb polymer in addition to increased blood oxygen-carrying capacity. In some studies it is showed that blood transfusion affects the pituitary-gonadal axis and leads to significant enhancement of the sperm parameter and improved fertility [5].

Diagnosis of SCD

There are several types of tests which are used to diagnose and monitor a sickle cell anemia (SCA) patient. Some of these tests are sickle cell test, complete blood count, hemoglobin (Hb) electrophoresis. In HbS, the complete blood count reveals Hb levels in the range of 6-8 g/dL with a high reticulocyte count [6].

Sensorineural deafness can be due to cochlear ischemia and anoxia that happened due to vessel occlusion secondary to the sickle RBC. The cochlea is highly sensitive to ischemia because of its anatomical position which is fed by a single artery called the labyrinthine artery that can be terminal [7].

Individuals suffering from SCD should be encouraged to have regular assessment of hearing function because of the high sensitivity of cochlea to develop reversible ischemia, especially during a vaso-occlusive crisis. Early detection of cholera ischemia and immediate intervention will prevent permanent hearing disability.

## Case presentation

A 43-year-old female with SCD since childhood has been on hydroxyurea. She had a frequent attack of a vaso-occlusive crisis with a history of avascular necrosis of both femoral heads and a history of osteomyelitis and autosplenectomy. She received multiple exchange transfusions and frequent blood transfusions complicated with iron overload and hepatic siderosis. She developed gestational cardiomyopathy with a reduced ejection fraction of 36%.

She was referred to the ENT clinic because she developed bilateral hearing loss for eight days more on the left side not associated with tinnitus, dizziness, or ear discharge. This condition was not preceded by an upper respiratory infection. Her condition was associated with generalized body pain and she was admitted to the hospital for sickle cell crisis and received IV hydration.

On examination there was no pre- or postauricular tenderness; her bilateral tympanic membranes were intact and there was no congestion or fullness. Weber's test showed Weber to the right ear. 

**Figure 1:**
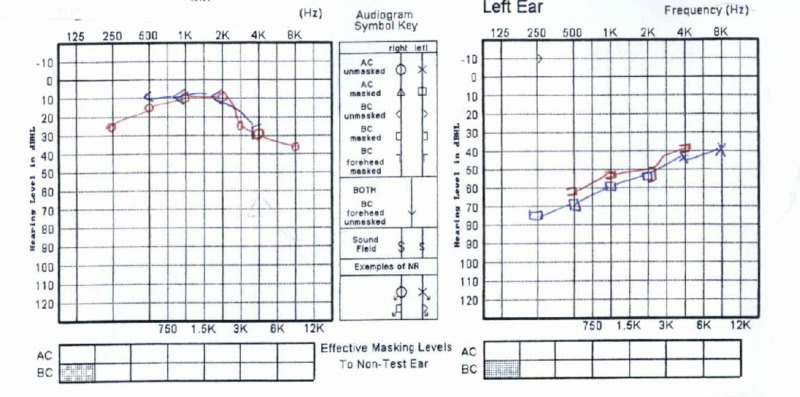
Audiogram results.

The hearing test was done: pure tone audiogram showed: see Figure 1.

Moderate to severe left sensorineural hearing loss (AB at 500 Hz + 4 kHz).

Right mild sensory neural hearing loss (AB at 250 Hz + 8 kHz).

Tympanogram showed: Type A bilaterally, Hb 5.9. 

She received IV fluids and analgesia and her condition drastically improved.

## Discussion

Sickle cell disease is the most common among black people. Hypoxia and dehydration are major precipitating factors for the sickling of RBCs. The structural changes that happened due to the sickling of RBCs reduce the capacity of RBCs to transport oxygen through the entire body [8].

Sudden onset sensorineural deafness is a rare complication of SCD. It could be unilateral or bilateral, mild or severe. The underlying mechanism of deafness is due to vascular occlusion because of the obliteration of the end terminal artery (Labyrnthine artery) that leads to cochlear damage. The tendency of cochlear to develop ischemia is very high, so specialized care and attention must be achieved to prevent hearing disability [9].

Multiple studies showed that underlying pathophysiology of cochlear impairment due to deformity of RBCs which blocks the blood supply to the high metabolic area consumes a high amount of oxygen to maintain the electrical balance of the endolymph. However, a lack of oxygen to the organ of Corti would cause extensive and severe cochlear damage that will explain the lack of otoacoustic emission in patients with SCD [10].

Meticulous literature review failed to explain the pathophysiology of inner ear complications in patients with SCD. However, the impairment blood supply of cochlea may be the root cause of these abnormalities. All hypotheses agreed that changes in cochlear blood supply in individuals with SCA initiate progressive local hypoxia that leads to cellular damage and permanent deafness [11]. 

There was a cross-sectional study done in 2018 which investigated the prevalence of sensorineural hearing loss in children and adolescents with SCA which showed that 28.8% of SCD exhibited sensorineural deafness [12].

Another study done in 2005 investigated 183 sickle cell subjects with a different variety of Hb electrophoresis (HbSS, HbSC, and S beta-thalassemia), each subject received a routine audiological and electrophysiological assessment. It showed 80% of the hearing impaired sickle cell subjects had Hb SS. The remaining hearing impaired sickle cell subjects were Hb SC 7% and Hb S beta-thalassemia 7% [13].

It seems from previous studies that there is a directly proportional relationship between the concentration of Hb S and the tendency to develop sensorineural deafness. So the major preventive measuring is by decreasing Hb S either by drug therapy like hydroxyurea or by exchange transfusion. In summary, the otologic complications of SCD in general and deafness, in particular, are not well understood and poorly studied.

## Conclusions

It is an obvious need to link SCD and sensorineural deafness and early diagnosis of hearing loss. Underdiagnosis or late diagnosis of hearing loss may lead to irreversible damage to the linguistic, biopsychosocial, and emotional development of patients who suffer from SCD.
